# Patients' preference for olanzapine orodispersible tablet compared with conventional oral tablet in a multinational, randomized, crossover study

**DOI:** 10.3109/15622975.2010.505663

**Published:** 2010-07-26

**Authors:** Istvan Bitter, Tamás Treuer, Nesrin Dilbaz, Igor Oyffe, Eda M Ciorabai, Severiano L Gonzalez, Sandra Ruschel, Jolanta Salburg, Yulia Dyachkova

**Affiliations:** 1 Department of Psychiatry and Psychotherapy, Semmelweis University, Budapest, Hungary; 2 Neuroscience Research, Eli Lilly & Company, Budapest, Hungary; 3 Ankara Numune Research and Training Hospital, Ankara, Turkey; 4 Lev-Hasharon Mental Health Center, Natania, Israel; 5 Spitalul Judetean Constanta, Romania; 6 Dr. Martinez Col Doctores, Monterrey, Nuevo Leon, Mexico; 7 Hospital Mario Kröeff-Serviço de Psiquiatria, Rio de Janeiro, Brazil; 8 Area Medical Center Vienna, Neuroscience Research, Eli Lilly & Company, Vienna, Austria

**Keywords:** Schizophrenia, olanzapine, patient preference, orodispersible tablet, oral conventional tablet

## Abstract

*Objectives*. The aim of this study was to compare patients’ preference for olanzapine orodispersible tablet (ODT) with oral conventional tablet (OCT). *Methods*. A 12-week randomized, crossover, multinational, open-label study was conducted to estimate the proportion of patients preferring ODT or OCT. Outpatients with stable schizophrenia on OCT monotherapy were randomly assigned 1:1 to ODT or OCT. Compliance and drug attitude were measured using the Drug Attitude Inventory (DAI-10) and Medication Adherence Form (MAF) scales; tolerability and safety by Association for Methodology and Documentation in Psychiatry (AMDP-5) questionnaire and adverse event summary. *Results*. A total of 175 patients answered a preference question: 106 (61%) preferred ODT and 48 (27%) preferred OCT (P<0.001 adjusted for treatment sequence); 21 (12%) expressed no preference. There was no significant change in DAI-10 with either formulation. MAF was above 75% in 94% vs. 93% of patients on ODC and OCT, respectively. Compliance as measured by tablet count was above 98% on both formulations. The adverse event profiles did not differ between formulations. Mean weight increase over 6 weeks on ODT was 0.8 kg and on OCT was 0.6 kg. *Conclusions*. Given the importance of patients’ preference for treatment planning and success, the ODT formulation should be routinely considered as a treatment option.

## Introduction

Active patient participation in therapeutic decision-making is generally viewed as a precondition to positive health outcomes (Street 2007). A greater understanding of patients’ preferences for new formulations of treatment is central to current models of shared patient-doctor decision making and has gained considerable interest in scientific research by applying open-label crossover, sequential trials (Voss and Klapper 2002; Dowson and Almqvist 2005; Nausieda et al. 2005; Slevin and Ryan 2006). Patients’ preference is both clinically and financially important, as it can have long-term implications in terms of patients’ motivation and insight into their disease state and its treatment, which might have a direct impact on the patient's compliance and treatment adherence (Kassirer 1994; Jahng et al. 2005). A recent systematic review (Preference Collaborative Review Group 2008) found that patients’ preference has an influence on study outcomes: patients who were randomized to their preferred treatment had a standardized effect size greater than those who were indifferent to their treatment assignments. However, some studies have not found a correlation between patient preferences and outcome (McKay et al. 1995; Ward et al. 2000). An analysis of treatment outcomes for depressed patients found that patients who received their preferred treatment option did better (mean difference in Beck Depression Inventory Score 4.6, 95% CI: 0.0-9.2) than those who were randomly assigned to treatment (Chilvers et al. 2001). The Clinical Antipsychotic Trial of Intervention Effectiveness (CATIE) also showed that more positive attitudes towards medication may improve outcomes (Mohamed et al. 2008). As patient preference for medication may influence outcomes, there is a need for patient preference studies in mental health research (Howard and Thornicroft 2006).

There is a small number of clinical trials where patients’ preferences for different formulations of the same drug have been studied. (Müller 2006). Questionnaire survey revealed that high preference for oral applications, particularly conventional tablets and capsules among psychiatric inpatients and staff members of a psychiatric department.

In other therapeutic areas, the preferences of patients with allergic rhinitis demonstrated the preference of the fast-dissolving tablet formulation (Roger et al. 2008). Another study concluded that capsule odour, difficulty swallowing, taste, breath odour had an impact on patients’ preference for choosing drug formulation of two types of cyclos-porine capsules (Steinberg et al. 2003).These results indicate that the characteristics of the formulation may play an important role in preference. However, patients’ acceptability and preference for the ODT relative to the OCT formulation of olanzapine has not been investigated.

In order to aid clinical decision making, this study aimed to determine the formulation preference of patients with schizophrenia regarding ODT and OCT and to elucidate factors associated with this preference. An open-label design was selected since blinding would eliminate the possibility for patients stating their preference for a specific formulation based on its physical characteristics.

## Study design

**Figure 1:**
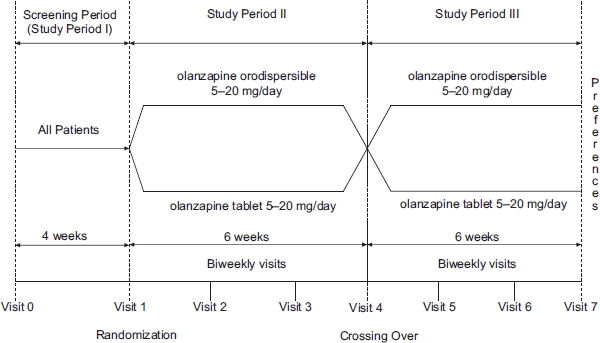
Study design: A 12-week, randomized, crossover, multinational, open-label study.

This 12-week, open-label, randomized, crossover multinational study was conducted to estimate the proportion of patients preferring ODT or OCT after 6 weeks of treatment with each formulation. The study started in May 2006 and was conducted in five countries (Brazil, Israel, Mexico, Romania and Turkey) at 19 investigational centers. All patients were outpatients, including patients in day hospitals. The study consisted of three periods (Figure 1). Period I was a 4-week screening phase for eligibility. At the beginning of period II, patients were randomly assigned in a 1:1 ratio to ODT or OCT at a dose of 5–20 mg in both arms. Randomization was using computer generated randomization sequence. The starting dose was at the discretion of the treating physician and could be adapted to patient needs during period II. At the start of period III, patients were switched to the alternative formulation (i.e. patients receiving ODT switched to OCT and patients receiving OCT were switched to ODT), but remained at the dose they received at the end of period II.

### Study population

Participants were eligible for the study, if they were aged 18–65 years, stable with respect to their symptoms of schizophrenia according to DSM-IV (APA 1994) or DSM-IV-TR (APA 2000) for at least 4 weeks before screening (visit 0), and able to take ODT or OCT formulations at dose 5–20 mg/ day as prescribed by the treating physician. All patients used OCT as antipsychotic monotherapy for at least 1 month before screening, and then continuously until randomization. For those patients who were using OCT before the randomization in doses higher/lower than allowed by the protocol, the dose was adapted in a range from 5–20 mg.

Patients were excluded from participation in the trial if previous treatment with olanzapine was inefficient and if they had a significant suicide risk, substance abuse or dependency. Also, patients with an unstable general medical condition, including history of seizures, uncorrected narrow-angle glaucoma, leu-copenia, diabetes, acute systemic infections, unstable cardiovascular disorders, with abnormal laboratory values or if they were human immunodeficiency virus positive, were excluded. Pregnancy and breastfeeding were also exclusion criteria. Patients having Clinical Global Impression of Severity (CGI-S) score (Guy 1976) >4 at Visit 0 and Visit 1; CGI-S score increase by one or more points between Visit 0 and Visit 1, or being considered as noncompliant with their previous antipsychotic medication according to physician opinion, were also excluded. Patients who were investigator site personnel directly affiliated with the study, or were immediate family of investigator site personnel and employees of the sponsor (Eli Lilly and Company) could not take part in the study.

This study was conducted according to the ethical principals stated in the Declaration of Helsinki. Ethical approval for the protocol was obtained from the institutional review board for each study site, and all participants (or equivalent appropriate legal authority) gave written informed consent for participation in the trial.

### Outcome measures

All patients who completed or discontinued the study after trying both formulations were asked to express their preference. This was the primary outcome variable and it was assessed by a three-choice question, which study investigators explored with their patients and recorded in the data report form. The question was formulated as: “Based on your patient's experience with both olanzapine forms in the study, which form of medication would your patient prefer to take in the future?” As an answer they could select: OCT or ODT, or refuse to express any preference. Patients were also asked to specify the reason for preference. There was no structured questionnaire for the preference reasons provided.

The patients’ subjective attitude to medication was measured by using the Drug Attitude Inventory DAI-10 scale (Hogan et al. 1983). Its total score comprises values from -10 to 10 with higher scores indicating more positive attitude towards medication. We used the score to examine how the attitude of patients with schizophrenia towards their medications may affect compliance. The DAI scale has been intensively used to investigate patient attitudes for oral antipsychotics (Hofer et al. 2002; Freudenreich et al. 2004; Day et al. 2005; Adewuya et al. 2006; Müller 2006) as well as on attitudes and preferences for oral with regard to depot medications (Heres et al. 2007; Patel et al. 2009). Patients’ clinical severity status was assessed using the Clinical Global Impression of Severity (CGI-S) scale for every study visit. Medication adherence was estimated by using the Medication Adherence Form (MAF) (Swartz et al. 2001). MAF is a single item “global judgment of medication adherence” completed by the person administering the scale, based on detailed questioning of the patient, or any other available source (e.g., family). Based on that information, the rater makes a global judgment of the time (almost never: 0-25%, sometimes: 26-50%, usually: 51-75%, almost always: 76-100%) the patient took the medication as prescribed since the last visit. Safety and tolerability of ODT versus OCT was measured by the AMDP-5 (Bobon and Woggon 1986). Standard laboratory tests measuring prolactin, liver function, blood biochemistry, whole blood count and ECG were performed. Furthermore, a physical examination of all patients was completed, which included height and weight. In order to follow up on appetite changes, a visual analogue scale (VAS) was used in all the visits starting from randomization (Gift 1989). The scale is a line with anchors at each end to indicate the extremes of the patient's hunger sensations in the study with values between 0 and 100. In our study, we measured appetite from most versus least and the patients had to check on the line where his/her appetite level was in the visual analogue scale.

### Statistical methods

The sample size for this study was calculated using the Mainland-Gart method (Senn 1993) for crossover trials based on a chi-square test that allowed for treatment sequence effects. The calculation was based on 80% power and 5% significance level as well as on the assumption of 60% preference for one of the two formulations and 10% rate for patients who express no preference or dropped out. The primary objective analysis utilized data from all patients without major protocol violations (per protocol set) who experienced both formulations on the study and expressed a preference for one of the two treatments received. The chi-square test determined whether treatment sequence and preference were associated, allowing to evaluate the preference adjusting for possible treatment sequence effects. Treatment sequence effect refers to the order in which the patients are receiving different formulations of the study medication. Patients who expressed no preference, or did not complete the final preference assessment, were excluded from the analysis. We also looked for an association between formulation preference and baseline patient characteristics: age, sex, ethnic origin, smoking status, type of diagnosis, duration of diagnosis, hospitalisation, family history, education, employment, marital and housing status, weight, CGI-S, DAI-10, VAS, MAF, and country effect using logistic regression models. Efficacy (CGI-S) was evaluated not only at endpoints but at each study visit and compared between the ODT and OCT patients using the repeated measurements ANCOVA model adjusted for treatment sequence and sequence by treatment interaction effect as well as MAF score. To better satisfy the assumptions of the analyses, the logarithmic transformation was applied to the CGI-S scores. The MAF and DAI-10 scores at endpoints were analyzed using a similar ANCOVA approach with adjustment for significant baseline covariates. Safety analyses were performed on all patients receiving at least one dose of study drug after randomization. Safety and tolerability was recorded by the AMDP-5 scale; in addition, VAS subjective appetite assessment, laboratory values, weight and vital sign measures were compared between ODT and OCT using ANOVAs with treatment sequence effect as a covariate. Non-normally distributed responses were log-transformed prior to analyses. The difference of weight and BMI from baseline to endpoint in the two treatment groups was calculated. The weight comparison was repeated for completers to ensure equal duration on each formulation and per weeks on study to adjust for duration effect in all patients. McNemar's test was used for the comparison of the number of patients with at least one adverse event (AE) between formulations. Study AEs were defined as any AE with the start date occurring after randomization.

### Study medications

Both olanzapine ODT and OCT formulations were used in this study as 5-, 10- or 20-mg tablets, administered orally once daily, preferably in the evening. Five to 20 mg/day of oral olanzapine dose is the documented effective range and it reflects the dose range in general clinical practice. ODT formulation's equivalence with the conventional olanzapine tablet is established and reported in the European Union Summary of Product Characteristics (EMEA Zyprexa Product Information 2008). Concomitant medications with primarily central nervous system activity (including other antipsychotics) were not allowed except for antidepressants other than fluvoxamine and monoamine oxidase (MAO) inhibitors and anti-convulsants, provided that a stable dose was given for at least 2 months prior to study entry and throughout study periods II and III. Concomitant medication use was recorded at each scheduled visit.

## Results

### Patients

**Figure 2:**
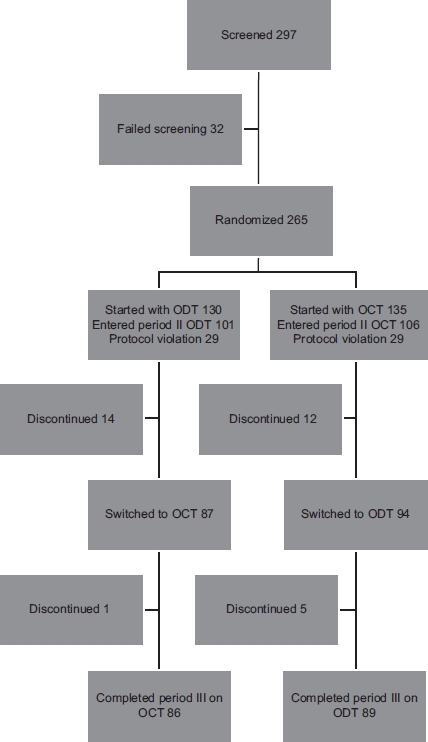
Patient flow diagram.

As presented in the patient flow diagram (Figure 2), 297 patients were screened, 32 failed screening, 265 patients from 5 countries (Brazil *[N=52],* Israel *[N=20],* Mexico *[N=49],* Romania *[N=42],* and Turkey [N= 102]) entered the study and were randomly assigned to ODT or OCT formulation. One hundred and thirty patients started with ODT formulation and 135 started with OCT formulation. Of 265 patients enrolled, 207 were included in the per protocol analysis set. Fifty eight patients (29 in each group) were excluded due to protocol violations. The major protocol violations were the following: no signed informed consent, unstable disease, patients not taking study medication or taking additional antipsychotic drugs, and taking ODT instead of OCD before the study. Out of 207 patients, 26 patients discontinued during study period II and six during period III. The most frequent reasons for discontinuation were lost to follow-up (13 patients), followed by sponsor decision (seven patients) and patient decision (five patients). The most frequent diagnosis was paranoid schizophrenia (160 [77%] patients) followed by undifferentiated schizophrenia (26 [13%]). Almost one-third of the patients (64 [31%]) had a history of hospitalization in the last 2 years, and 78 (38%) patients had a family history of schizophrenia. A high proportion of patients in the ODT group (41 [41%]) and in the OCT group (48 [45%]) entered the study with pre-existing conditions, where obesity was the most common (16 [16%] patients and 24 [23%] patients in ODT and OCT groups, respectively). Further baseline characteristics of the per-protocol analysis set are presented in Table I.

**Table I:** Demographic characteristics of patients in primary and secondary analyses

	All patients	Started from ODT	Started from OCT
Total, *N* (%)	207 (100)	101 (100)	106 (100)
Sex, male, *N* (%)	131 (63.3)	61 (60.4)	70 (66.0)
Caucasian, *N* (%)	131 (63.3)	66 (65.3)	65 (61.3)
Hispanic, *N* (%)	54 (26.1)	24 (23.8)	30 (28.3)
Other, *N* (%)	22 (10.6)	11 (10.9)	11 (10.4)
Age, mean ± SD	35.3 ± 11.1	35.2 ± 10.4	35.5 ± 11.8
Weight, kg, mean ± SD	75.0 ± 14.9	73.2 ± 14.3	76.8 ± 15.3
BMI kg/m2, mean ± SD	26.9 ± 5.0	26.5 ± 5.0	27.3 ± 4.9

### Treatment patterns and concomitant medications

Patients had to use OCT formulation (as monother-apy) for at least 1 month before the study entry to be eligible to enter screening for the trial. Retrospective medication history showed that, during the last 2 years, patients used a variety of medication treatments before they started with the OCT formulation. Forty-four (21%) patients had no previous antipsy-chotic treatment for the past 2 years prior to the olanzapine treatment; four (2%) patients were using olanzapine mono therapy, fifteen (7%) used olanzapine and one other medication; 77 (37%) patients used one non-olanzapine medication only; 66 (32%) patients used two or more non-olanzapine medications; and one patient was never treated in the past prior to current olanzapine treatment. At study entry (visit 0), all 207 (100%) patients were on olanzapine treatment, of which 173 (84%) were on olanzapine monotherapy.

The mean dose throughout the study was 12.3 mg/day for ODT and 12.4 mg/day for OCT. Forty-eight (24%) patients were receiving the maximum dose allowed of 20 mg/day. Information about concomitant medications was collected at each of the three study periods. During the first treatment period (visits 1 to 4), there were 12 (12%) patients in the ODT group and 20 (19%) patients in the OCT group using concomitant medications. In the ODT group, all 12 patients used 12 different medications, including diazepam, bromazepam, capto-pril and carbamazapine, among others. The most commonly used concomitant medications in the OCT group were diazepam (5 [4.7%]), glibencl-amide (2 [1.9%]), valproic acid (2 [1.9%]), hydro-chlorothiazide (2 [1.9%]), and simvastatin (2 [1.9%]). After the crossover (visits 4-7), in the group changing from OCT to ODT, 19 (19%) patients continued concomitant medications, and 11 (10.4%) took concomitant medications in the group changing from ODT to OCT.

### Preference results

Significantly more patients preferred ODT over OCT. One hundred and seventy-five patients answered the preference question: 85 patients from the group which started with ODT formulation and 90 from the group which started with OCT formulation. Overall, 106 patients (61%) preferred ODT and 48 (27%) preferred OCT (p<0.001 adjusted for treatment sequence); 21 (12%) patients expressed no preference. Patient preference by treatment periods are provided in Table II. None of the baseline factors explored had a statistically significant association with the formulation preference expressed.

**Table II:** Patient preference for olanzapine formulation by treatment sequence.

		Started from	Started from
	All, N (%)	ODT, N (%)	OCT, N (%)
Total preference	175 (100)	85 (100)	90 (100)
Preference period II	55 (31.4)	40 (47.1)*	15 (16.7)*
Preference period III	99 (56.6)	33 (38.8)*	66 (73.3)*
No preference expressed	21 (12.0)	12 (14.1)	9 (10.0)

* *P*<0.001 from chi-square test for preference by treatment sequence association indicates difference in preference between formulations. Patients who expressed no preference were excluded from the test.

As a secondary finding, it was observed that a higher percentage of patients (57%) would prefer to take, in the future, the formulation from period III rather than switch back to the formulation from period II. If patients start with ODT, only 47% preferred ODT formulation versus 39% who preferred OCT indicating the shift from overall preferences towards the last formulation. Similarly, of the patients who started with OCT, only 17% preferred OCT and 73% preferred ODT indicating similar shift towards the last formulation. If there was no sequence effect independently from what they started with, similar proportions would prefer the same formulation (about 27% OCT and 61% ODT). But even when ODT was taken first during the crossover study, the preference for it was higher than for OCT at the end of the study. In summary, these data suggest that the impact of the experience of patients on the last formulation taken was not strong enough to overcome the formulation preference results.

### Secondary measures

Patients had to be stable on olanzapine to be eligible for the study. Disease severity, measured by CGI-S, could not increase and had to remain as 4 points or less during screening to be eligible for randomization. CGI-S remained stable throughout the study: at randomization mean CGI-S for ODT was 2.3 [Standard Deviation (SD) 0.75]; for OCT 2.5 [SD 0.73], at the end of the first 6 weeks period mean CGI-S for ODT remained at 2.3 [SD 0.73] and for OCT 2.4 [SD 0.76]. After the switch to the other formulation, patients remained stable: at the end of the study in the group which changed from OCT to ODT, the mean CGI-S was 2.4 [SD 0.72] and in the group which changed from ODT to OCT mean CGI-S was 2.2 [SD 0.81]. Results of a repeated measures ANCOVA, adjusted for compliance (MAF), did not indicate any difference in efficacy between treatment formulations (P=0.87). No significant change in subjective experience with the medication was found as measured by DAI-10: mean increase 0.2 on ODT [SD 1.76], 0.0 on OCT [SD 1.57]. The ANCOVA adjusted for patient baseline characteristics did not indicate a difference between treatment formulations (P=0.29). A medication adherence above 75% as measured and defined by MAF scale was found in 182 (94%) versus 179 (93%) patients on ODC and OCT, respectively. No patients were less than 50% adherent at any time on any formulation. Compliance measured by tablet count was above 98% for both formulations. Housing status had the strongest association with compliance (P<0.001): compliance in patients in supervised residence or being homeless was lower than in patients with independent residence. Free text comments about the preference reasons for both formulations were collected. Common reasons for preference of a specific formulation, as indicated by patients, were ease of use, taste of the formulation, expectation of better effectiveness and weight change.

### Safety results

The safety population included all the patients who received at least one dose of study medication at some time in the study. Two-hundred and fifty patients who received ODT treatment and 248 patients who received OCT treatment were analyzed for the safety data. The total number of patients with at least one AE while treated with ODT was 42 (16.8%) and with OCT 31 (12.5%) (P=0.31) (Table III). Thirty-two (12.8%) patients receiving ODT treatment and 24 (9.7%) receiving OCT treatment (P=0.33) experienced at least one AE related to study drug (as described by investigators). The most common adverse events in the safety population were: weight increase (ODT 19 [7.6%]; OCT 15 [6.0%]), somnolence (ODT 4 [1.6%]; OCT 5 [2.0%]), and, hypertriglyceridemia (ODT 6 [2.4%] patients; OCT 3 [1.2%] patients). Mean weight increase over 6 weeks in the ODT group was 0.8 kg [SD 2.29] and in the OCT group 0.6 kg [SD 1.97].There were two serious adverse events (SAEs) reported in this study: appendicitis and completed suicide. None of those SAEs were considered as related to study drug by the investigators. According to the AMDP-5 scale, safety and tolerability did not differ between formulations. Changes in appetite as measured by the visual analogue scale (VAS) did not reveal a consistent pattern (mean change in ODT -0.1 [SD 14.9]; in OCT 2.0 [SD 14.7]).

**Table III:** Number of patients with adverse events by treatment and study period (safety population).

	Started from ODT		Started from OCT		Pooled by treatment	
AEs by MedDRA version 9.1	Period II (ODT)	Period III (OCT)	Period II (OCT)	Period III (ODT)	ODT	OCT
Total patients treated, *N* (%)	130 (100)	116 (100)	135 (100)	120 (100)	250 (100)	248 (100)
Total patients with at least one AEs, *N* (%)	27 (20.8)	7 (6.0)	24 (17.8)	15 (12.5)	42 (16.8)*	31 (12.5)*

* *P*=0.3

## Discussion

Most of the patients who answered the preference question declared they preferred ODT over OCT. None of the baseline factors explored (age, sex, origin, smoking status, type of diagnosis [paranoid vs. others], duration of diagnosis, recent hospital-ization, family history, education, work, marital status, housing status, baseline weight and BMI, drug attitude, VAS scale [appetite scale], compliance, or country of residence) had a statistically significant association with the formulation preference expressed. The generalizability of the results is limited to stable adherent patients with schizophrenia without substantial comorbid issues who can do well on olanzapine monotherapy between doses of 5-20 mg/day; however, it was important to test formulation preference in stable patients to avoid interaction of their judgement with symptom improvement. Although data suggesting that patient preference can be correctly and reliably measured by a single preference question, this method is not a standardized measure.

This is the first multicenter, large-scale, randomized trial in psychiatry to assess preferences for these two oral formulations of olanzapine in patients who were treated in a manner similar to common clinical practice. The open-label, crossover design is an accepted or established method in the literature to compare formulation preference. There is data suggesting that patient preference can be correctly and reliably measured by a single preference question (Allain et al. 2003).

The effectiveness of OCT in the treatment of patients with schizophrenia is widely investigated in several randomized, controlled trials and in observational studies as well, for example, the Effectiveness of Antipsychotic Drugs in First-Episode Schizophrenia and Schizophreniform disorder (EUFEST) trial (Kahn et al. 2008), the CATIE trial (Lieberman et al. 2005), the Schizophrenia Outpatient Health Outcomes studies (Novick et al. 2007; Bitter et al. 2008; Dossenbach et al. 2008) and in a recent meta-analysis (Leucht et al. 2009). In recent years, more clinical attention has been paid to oral dis-persible formulation of medications including ODT. Pharmacokinetic studies have shown that olanzapine ODT is bioequivalent to OCT with the same rate and extent of bioavailability (Bergstrom et al. 2004).

Moreover, the orodispersible formulation of olanzapine is well accepted and effective in acute settings and demonstrated significant improvement in Positive and Negative Symptoms Scale (PANSS) total score in a 6-week open-label study with acutely ill psychotic patients who used ODT formulation (Kinon et al. 2003). In this trial, by week 6, 60% of patients were considered responders and the study authors concluded that the ODT formulation of olanzapine was effective in the rapid reduction of psychopathology. In an observational study, two patient groups receiving ODT or OCT showed similar effectiveness after 2 weeks (Czekalla et al. 2007). Another study confirmed the effectiveness of ODT formulation as measured by Positive and Negative Symptoms Scale Excitement Component score (PANNS-EC) for the treatment of acute agitated patients in a naturalistic, open-label study (Pascual et al. 2007). A study by Dardennes et al., with 62.4% of poorly compliant or noncompliant patients, concluded that those patients who received the ODT formulation had an effective reduction of psychotic symptoms (Dardennes et al. 2004). An outpatient study in schizophrenia or schizoaffective disorder (*n*=174) showed that use of the ODT formulation could increase compliance (Dilbaz et al. 2006).The recent review of ODT formulation publications (San et al. 2008) confirmed that orally disintegrating olanzapine is an effective atypical antipsychotic with an acceptable safety profile and can reduce the burden of treatment on patients and caregivers due to its ease of administration. Although most patients preferred ODT over OCT in our trial, our study did reveal an association between preference and treatment sequence whereby patients more frequently indicated that they would rather continue the current formulation than go back to the previous one. The fact that only 12% of patients did not show preference to the different formulations suggests that preference can be assessed in clinical settings and formulation does matter to patients.

The main objective of the study was to compare patients’ preference for the available oral formulations of olanzapine, so the study was not powered to address the secondary objectives such as differences between formulations in outcomes such as CGI-S, compliance, drug attitude, weight, or others. It is still an open question whether this short-term preference favoring the ODT formulation could result in better adherence in longer-term treatment. The study was an open-label randomized study, as blinding was not appropriate since preference was related to the physical characteristics of the formulations themselves. Collected and grouped reasons might reflect patients’ expectations rather than ease of use and taste of the formulation, but the former information cannot be analysed statistically since the question was not designed accordingly and answers, therefore, represent a collection of single personalized reasons. It was also a possible ground for bias that the formulation considered to be more technologically advanced was also preferred and perhaps this perception, if it existed, may also explain the preference for ODT in the current study.

Although several papers and communications have reported possible weight reduction or less weight gain when patients start or switch to ODT, as contrasted with OCT (Karagianis et al. 2008), our study did not find meaningful differences in this regard. However, this study was not powered to detect weight differences.

## Conclusion

Most of the patients who answered the preference question declared they preferred olanzapine orodispersible over olanzapine conventional formulation. The olanzapine orodispersible tablet formulation should be considered a useful alternative to classical conventional olanzapine in appropriate patients, if patient preference is taken into account. The impact of formulation preference on long-term adherence warrants further investigation.

## Statement of interest

This study was sponsored by Eli Lilly and Company. In compliance with the Uniform Requirements for Manuscripts, established by the International Committee of Medical Journal Editors, the sponsor of this study did not impose any impediment, directly or indirectly, on the publication of the study's results. This research was funded by Eli Lilly and Company, Indianapolis, USA. Employees of Eli Lilly and Company were involved in the study design, in the collection, analysis and interpretation of data, in the writing of the manuscript, and in the decision to submit the manuscript for publication.

Tamas Treuer, Yulia Dyachkova, Jolanta Salburg are employees of Eli Lilly and Company. Istvan Bitter has been an advisory board member/consultant/lecturer for AstraZeneca, Bristol-Myers Squibb, Eli Lilly, EGIS, Janssen, Lundbeck, Novartis, Pfizer, Richter and ScheringPlough. Eda Maliche Ciorabai received honorariums by Eli Lilly, Pfizer, Sanofi, AstraZeneca for consulting works. Nesrin Dilbaz has been consultant for Pfizer, Janssen-Cilag, Servier, Lilly, Solvay; participated in research, for Lundbeck, Janssen-Cilag, Servier, Pfizer, Eli Lilly, AstraZeneca, BMS and lectured for Lundbeck, Janssen-Cilag, Servier, Pfizer, AstraZeneca, Glaxo-SmithKline, Solvay, Eczacibasi-Zentiva. Igor Oyffe worked as researcher in projects sponsored by Eli Lilly.
